# Deciphering Redox State for a Metal-Rich World

**DOI:** 10.1007/s11214-022-00872-9

**Published:** 2022-03-01

**Authors:** Timothy J. McCoy, Steven D. Dibb, Patrick N. Peplowski, Clara Maurel, Hannah L. Bercovici, Catherine M. Corrigan, James F. Bell, Benjamin P. Weiss, David J. Lawrence, Daniel D. Wenkert, Thomas H. Prettyman, Lindy T. Elkins-Tanton

**Affiliations:** 1 Dept. of Mineral Sciences, National Museum of Natural History, Smithsonian Institution, Washington, DC 20560-0119 USA; 2 School of Earth and Space Exploration, Arizona State University, Tempe, AZ 85287 USA; 3 Johns Hopkins Applied Physics Laboratory, Laurel, MD 20723 USA; 4 Dept. of Earth, Atmospheric and Planetary Sciences, Massachusetts Institute of Technology, Cambridge, MA 02139 USA; 5 Mission Systems and Operations Division, Jet Propulsion Laboratory, California Institute of Technology, Pasadena, CA 91109 USA; 6 Planetary Science Institute, Tucson, AZ 85719 USA

**Keywords:** Psyche, Oxidation-reduction, Iron meteorites, Chemical composition, Spectroscopy

## Abstract

The Psyche mission’s Oxidation-Reduction Working Group is focused on understanding, determining, and applying the redox state of (16) Psyche to understand the origin of a metal-rich world. The oxidation-reduction state of an asteroid, along with its temperature, parent body size, and composition, is a key parameter in determining the history of an asteroid. Determining the redox state from spacecraft data is most easily done by examining potential metal-oxide buffer pairs. The occurrence of Ni, Fe, C, Cr, P and Si, in that order, in the metal or sulfide phase of an asteroidal body indicates increasingly reduced conditions. Key observations by the Imager and Gamma-Ray and Neutron Spectrometer (GRNS) of Psyche can bracket the redox state using metal-oxide buffers. The presence of Fe,Ni metal can be confirmed by the ratios of Fe/O or Fe/Si and the concentration of Ni variability in metal across the asteroid can be determined by GRNS. The FeO concentration of silicates is complementary to the Ni concentration of metal and can be constrained using filters on the Imager. The presence of FeO in silicates from ground-based observations is one of the few measurements we already have of redox state, although available data permit a wide range of silicate compositions and mineralogies. The presence of C, P or Si concentrated in the metallic, Fe-rich portion of the asteroid, as measured by GRNS, or Ca-sulfide, determined by imaging, would indicate increasingly reducing conditions. Linkage to known types of meteorites, whether metal-rich chondrites, stony-irons or irons, expands the mineralogical, chemical and isotopic data not available from remote observations alone. Redox also controls both silicate and metal mineralogy, influencing differentiation, solidification, and subsolidus cooling, including the relative abundance of sulfur in the core and possible magnetic signatures. The redox state of Psyche, if a fully-differentiated metallic core, might constrain the location and timing of both the formation of Psyche and any oxidation it might have experienced.

## Introduction

The instrument suite of the Psyche spacecraft will measure the chemical composition, provide constraints on the mineralogy, measure the magnetic signature, and determine the mass and size of the asteroid. These observations are key to understanding the history of (16) Psyche, ranging from processes occurring today such as space weathering, to those that occurred 4.6 billion years ago including the formation of the chondritic protolith and, possibly, the differentiation of the asteroid. One goal of the mission is to relate the history of Psyche to that of the terrestrial planets to understand shared features and histories.

As early as Prior (1916), oxidation-reduction (redox) reactions were recognized as a fundamental control on the mineralogy and mineral compositions of meteorites. Prior’s rule states that when less metal is present in meteorites, the existing metal is richer in nickel. In addition, the associated silicates are richer in FeO. In essence, Prior (1916) envisioned meteorites, particularly the undifferentiated chondritic meteorites, as isochemical, with redox as the major control. This view was widely accepted until the work of Urey and Craig (1953), who established that different meteorite groups were, in fact, not isochemical. Nonetheless, chondritic meteorites, considered the precursor to differentiated planetary objects, exhibit a wide range of oxidation states (Righter et al. 2016). Likewise, the terrestrial planets range widely in oxidation state from the highly reduced Mercury to the relatively oxidized Mars. The difference in the redox state of elements – particularly for iron – is found in bodies ranging from undifferentiated chondrites to fully differentiated planets. Thus, the difference is a property likely established in the precursor chondrite and inherited during differentiation. Redox state variation within meteorites and terrestrial planets served as the impetus for our effort to constrain the oxidation state of Psyche.

In this chapter, we explore the potential role of redox on the formation of Psyche. For clarity, the use of “Psyche” refers to the asteroid, whereas the terms “spacecraft”, “mission” and “project” are used for those aspects of the mission. We begin with an exploration of redox – what is it, how do we quantify it, and how does it control element partitioning between minerals (e.g., silicates, metal)? In the latter part of the chapter, we discuss how the mission team, specifically the Oxidation-Reduction Working Group, will use data from the spacecraft’s instruments to constrain the oxidation state of the asteroid. Finally, we address how redox-controlled mineralogy influences the history of a body and may help us constrain the origin of Psyche.

## Oxidation-Reduction: A Primer for Psyche

From a chemical perspective, oxidation is the loss of electrons by an element and, conversely, reduction is the gain of electrons by an element. A useful example is the combustion of carbon in the presence of oxygen:

Reaction 1 $$\begin{aligned} \text{C} + \text{O}_{2} = \text{CO}_{2} \end{aligned}$$

In this reaction, carbon is being oxidized from 0 to 4+, whereas oxygen is being reduced from 0 to −2. A useful way to think of this is that carbon bonds with oxygen during combustion, transforming from the reduced elemental state to the oxygen-bearing state. As with all redox reactions, the reduction of one element is balanced by the oxidation of another. This reaction – and its variant to produce carbon monoxide – are both societally relevant with the mass burning of fossil fuels contributing to the increase in atmospheric CO_2_ and to understanding Psyche. The reverse of this reaction – transformation of CO_2_ to carbon – occurs during photosynthesis through a complex set of reactions occurring during the Calvin cycle which incorporate CO_2_ from the atmosphere into glucose. As carbon comprises about 50% of the dry mass of trees, about half the mass of the largest tree you have ever seen materialized out of thin air during photosynthesis!

To quantify the redox state, geologists typically use oxygen fugacity, which is an equivalent of the partial pressure of oxygen in a particular environment, most commonly rocks, corrected for the nonideal behavior of the gas. An instructive example is the oxygen fugacity of air. At 21% O_2_, the oxygen fugacity of air is $\sim10^{-0.7}$. Oxygen fugacities are typically plotted on diagrams of log (ƒO_2_) vs. 1/T (K). Thus, air plots at about −0.7. One of the non-intuitive parts of oxygen fugacity is that it measures the partial pressure of gaseous oxygen, rather than the concentration of oxygen that might be bound in a rock. Thus, rocks, which typically have 40–50 wt.% oxygen plot many orders of magnitude lower in oxygen fugacity than air, which contains 21 wt.% oxygen.

To circumvent the difficulty of comparing temperature dependent oxygen fugacity values for various geological equilibria, pairs of metal and oxide for the same element are commonly used. At constant pressure and temperature, the *co-existence* of metal and oxide fixes the ƒO_2_ at a specific value, such that an increase or decrease in the *amount* of oxide or metal will not change the ƒO_2_. Hence the use of the word “buffer” or “oxygen buffer” for these metal-oxide pairs. Oxygen fugacities for these metal-oxide pairs are often determined by electrochemical or calorimetric means (e.g., Sato 1971). The slope of these buffer curves in log ƒO_2_ − 1/T space is negative (the ƒO_2_ decreases with decreasing temperature), because of the relationship between ƒO_2_ and free energy (G). As most buffers are metal and oxide, their free energy and thus their slopes are similar, making their use convenient for a temperature-independent comparison (Ellingham 1944). In addition to removing the strong dependence of ƒO_2_ on temperature, they have the decided advantage when working with spacecraft data that the occurrence of an element as a metal – either in a metallic phase or a sulfide – can constrain the oxygen fugacity relative to the metal-oxide buffers. Here we discuss several metal-oxide buffers relevant to our investigation of Psyche, including iron, nickel, carbon, phosphorus, chromium and silicon.

For the benefit of those unfamiliar with meteorite nomenclature used here (Weisberg et al. 2006), individual meteorites are given names based on the site where they fell or were found (e.g., Canyon Diablo, Arizona). Undifferentiated meteorites, called chondrites, are divided into classes (e.g., C for carbonaceous, E for enstatite) and groups and given letter designations (e.g., H for high-iron ordinary chondrites, CB for carbonaceous chondrites similar to the type specimen Bencubbin). Differentiated meteorite groups (e.g., eucrites, brachinites) either have historical names (e.g., eucrite from the Greek referring to the easily distinguished mineral grains) or are named after the type specimen (e.g., brachinites from Brachina, Australia). Iron meteorites are subdivided on the basis of chemical composition – originally Ga, Ge and Ni – using a Roman numeral (I-IV) and letter (A-G). In some cases, groups thought to be distinct (e.g., IIA and IIB) were found with analyses of additional meteorites to be end members of a common group (e.g., IIAB). Meteorites within a type (e.g., H, CM, IIAB) are thought to have originated on a common parent body.

### Iron

With valence states of 0, 2+ and 3+, iron is one of the most widely used elements in metal-oxide buffers, as it covers a wide range of oxidation states applicable from the interior (core, mantle) to the surface (crust) of the Earth. Under highly oxidizing conditions, a buffer exists between magnetite ($\text{Fe}^{2+}\text{Fe}^{3+}_{2}\text{O}_{4}$) and hematite ($\text{Fe}^{3+}_{2}\text{O}_{3}$). This buffer is seldom applied in meteorites as most hematite likely forms by alteration of metal in Earth’s oxygen-rich atmosphere. At conditions slightly more reducing is a buffer between FeO and $\text{Fe}^{2+}\text{Fe}^{3+}_{2}\text{O}_{4}$. FeO is commonly called wüstite after the mineral of that formula ($\text{Fe}_{(1-x)}\text{O}$). The wüstite-magnetite buffer is likewise seldom invoked, as geologists tend to prefer a mineral buffer reaction between quartz, fayalite and magnetite, commonly termed FMQ:

Reaction 2 $$\begin{aligned} 3 \text{Fe}_{2}\text{SiO}_{4}\ (\text{fayalite}) + \text{O}_{2} = 2 \text{Fe}_{3}\text{O}_{4}\ (\text{magnetite}) + 3 \text{SiO}_{2}\ (\text{quartz}) \end{aligned}$$

Magnetite is present in some chondritic meteorites (e.g., CK, CI, CM), typically because of aqueous alteration of metal on the parent asteroid early in the history of the Solar System, not as a result of formation in an oxidizing region of the nebula.

**Fig. 1:**
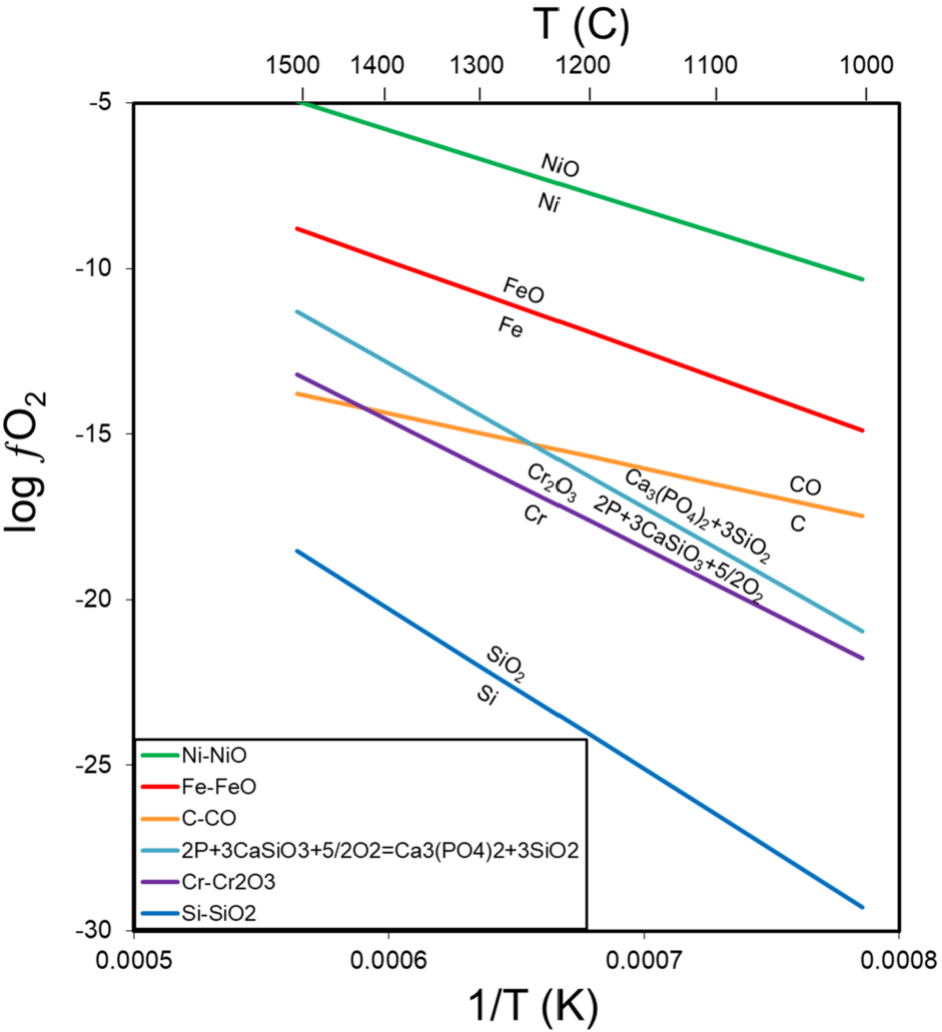
Log ƒO_2_ vs 1/T (K) for metal-oxide or mineral buffers in the range of $1000\text{--}1500~^{\circ}\text{C}$ for Ni, Fe, C, P, Cr and Si using data from O’Neill and Pownceby (1993) (Fe, Ni), Holzheid and O’Neill (1995) (Cr), French and Eugster (1965) (C), and Robie et al. (1978) (P, Si). Among these elements, Si is the most readily and Ni the least readily oxidized

Most relevant to Psyche is the iron-wüstite (IW) buffer (Fig. 1), in large part because iron is certainly the most abundant element in any asteroid core. The position of the buffer was calculated using electrochemical data from O’Neill and Pownceby (1993) over the range of temperatures ($1000\text{--}1500~^{\circ}\text{C}$) at which melting, differentiation and core formation occur on asteroids. At this buffer, iron metal (Fe) coexists with iron oxide (FeO), typically in silicates (olivine, (Mg,Fe)_2_SiO_4_; pyroxene (Mg,Fe)SiO_3_). For an oxygen fugacity above this buffer, iron metal is unstable, but below the buffer, FeO in silicates decreases with increasing reduction but remains present until extremely reducing conditions, a topic we discuss later. Oxygen fugacity is typically reported relative to this buffer, so IW+1 is one log unit above the IW buffer and IW-2 is two log units below the IW buffer. The identification of indigenous metallic iron, even in the presence of FeO, on a planetary body constrains the oxygen fugacity to at or below the IW buffer.

**Fig. 2:**
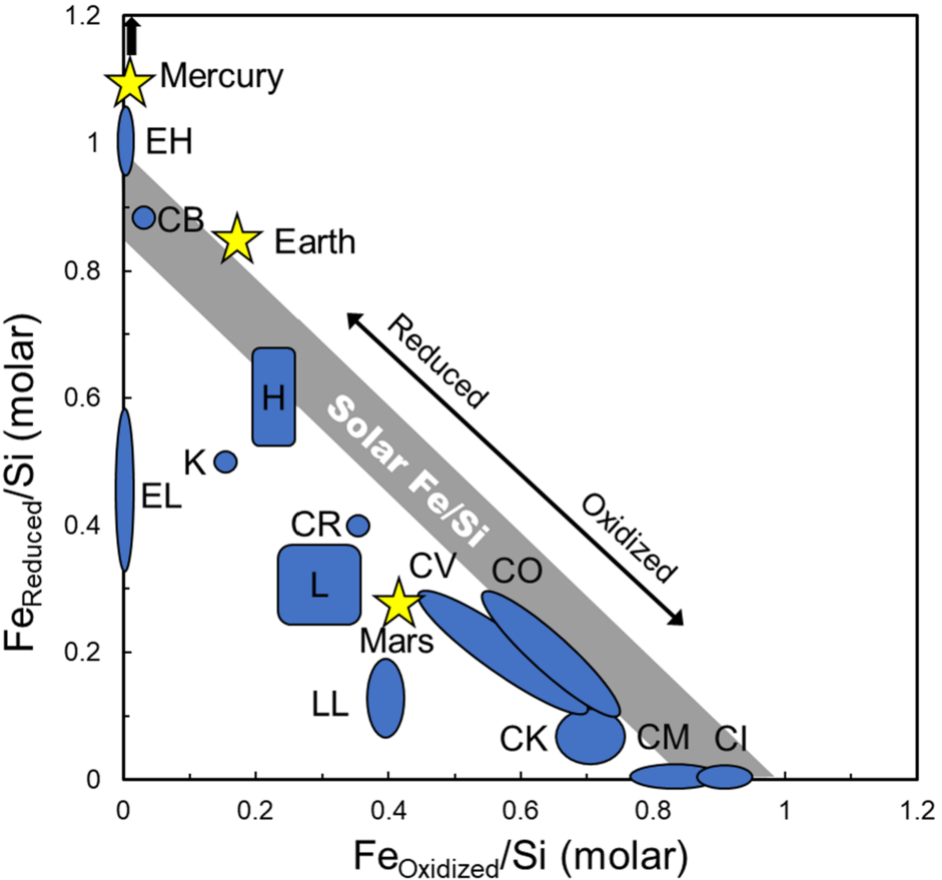
Modified Urey-Craig plot of Fe_oxidized_/Si vs Fe_reduced_/Si (molar), after Yoshizaki et al. (2021) with additional data for the CB chondrite Bencubbin (Jarosewich 1990), Mars (Yoshizaki and McDonough 2020) and Mercury (Nittler et al. 2018). The Fe_reduced_/Si ratio of CB chondrites likely is from a sample depleted in metal relative to the bulk meteorite. Note that data for CH and G chondrites is not available, but they likely plot close to CB chondrites. Redox control can vary a composition along a tie line of total Fe/Si, such as the Solar Fe/Si ratio, whereas differences in total Fe/Si, such as between H, L and LL ordinary chondrites, cannot be redox controlled

The redox relationship between Fe in metal and sulfides, collectively grouped as reduced iron, and FeO in silicates and oxides, grouped as oxidized iron, in meteorites was the impetus behind one part of Prior’s rule – the less metal, the more FeO in the associated silicates. Urey and Craig (1953) would use superior wet chemical analyses of ordinary chondrites which measured reduced iron in metal and sulfides and oxidized iron in silicates and oxides, as well as total iron, to demonstrate that two groups of ordinary chondrites, called H and L chondrites, differed in total iron and must sample distinct geochemical reservoirs. The Urey-Craig diagram (Fig. 2) serves to illustrate the differences in redox between different chondritic meteorites and planetary bodies and provides a useful conceptual framework for observations of the relative abundance of reduced and oxidized iron on Psyche. Meteorite groups plotting at high reduced iron and low oxidized iron values, including the EH and EL enstatite chondrites and the metal-rich CB chondrites (and, likely, the closely related CH and G chondrites, for which data is unavailable), have iron largely in the reduced state, with low FeO in silicates. In contrast, meteorite groups plotting at high oxidized iron and low reduced iron, including CI and CM chondrites, have little iron in the reduced state with FeO-rich silicates and, typically, magnetite. Oxidation-reduction moves compositions along a constant total Fe/Si ratio, such as shown by Solar Fe/Si. Thus, from the perspective of total Fe/Si, H and CO chondrites, neither of which experienced significant aqueous alteration, are close to isochemical but differ in their proportions of oxidized and reduced iron. Different total Fe/Si ratios cannot be related simply through redox control, as redox does not change the total abundance of iron relative to silicon, but result from other processes including accretion and, in the case of asteroids, later impact. Compositions of planets are less constrained than meteorite groups with considerably larger uncertainties, but illustrate increasing oxidation (and, interestingly, decreasing total Fe/Si) and decreasing core size from Mercury ($\sim70\%$ of total radius) to Earth (32%) to Mars (21%) (e.g., Righter et al. 2006). Interestingly, the biggest uncertainty in planetary compositions is often the size and composition of the core, whereas if Psyche is a core, it may offer the inverse problem of a chemically well-characterized core possibly with remnants of the less well constrained mantle or crust.

### Nickel

As the second most abundant element in meteoritic metal, with concentrations ranging from 5–60 wt.% in iron meteorites, nickel exhibits significant variation both within and between iron meteorite groups. The Ni-NiO buffer from $1000\text{--}1500~^{\circ}\text{C}$ calculated using electrochemical data from O’Neill and Pownceby (1993) plots from 4.6 to 3.8 log units above Fe-FeO (IW) (Fig. 1). Thus, Fe in the metallic form will oxidize much more readily than Ni in the metallic form. Therefore, oxidation, even at conditions below the IW buffer, will enrich the residual metal in Ni relative to Fe. At conditions more oxidizing than IW, Ni is incorporated into sulfide phases like pentlandite. This is the case for the CK chondrites, which lack metallic iron. Later in this chapter, we discuss the role of redox reactions in establishing the differences in composition between groups of iron meteorites.

### Carbon

Carbon occurs in many meteorites, often as soluble or insoluble organic matter in carbonaceous chondrites or as graphite in enstatite chondrites, some groups of achondrites (ureilites, acapulcoites-lodranites, winonaites) and iron meteorites (notably the IAB irons). Graphite typically occurs with relatively FeO-poor silicates in meteorites. Not surprisingly then, the C-CO buffer (French and Eugster 1965) occurs at conditions more reducing than the IW buffer (Fig. 1). This fact has been exploited for centuries when coke (coal heated in an oxygen-poor atmosphere) is mixed with iron oxides during the smelting of iron metal. Thus, a finding of indigenous graphite in an asteroidal core would indicate reducing conditions. A more interesting feature of the C-CO buffer is its slope on a plot of log ƒO_2_ vs 1/T (K) (Fig. 1). Most metal-oxide buffer pairs are subparallel, as observed with Fe-FeO and Ni-NiO. In contrast, C-CO differs dramatically in slope from other metal-oxide buffers. At $1000~^{\circ}\text{C}$, C-CO is IW-2.6, but is IW-5.0 by $1500~^{\circ}\text{C}$. This slope results from the fact the reaction contains both a solid (C) and a gas (CO), whereas other buffers are solid-solid buffers (e.g., Fe-FeO). Thus, while graphite can serve as a reductant for iron at the entire relevant range of temperatures, it can also serve as a reductant for elements like Cr and P at higher temperatures. Modern industrial processes exploit this by mixing carbon and silica sand at extremely high temperatures to produce silicon metal as an input product for the manufacturing of silicon wafers for computers. Finally, the solid-gas nature of the C-CO buffer results in it being particularly pressure sensitive, shifting by $\sim4$ log units to more oxidizing conditions (closer to IW) with a pressure increase from 1–100 bars (Brett and Sato 1984; Walker and Grove 1993; Benedix et al. 2005). This has interesting consequences in a C-buffered system if, for example, the mantle of a partially molten asteroid was stripped resulting in a lower pressure in the now exposed core.

### Chromium and Phosphorus

These two buffers are similar enough – and serve to illustrate the same geochemical behavior – that we treat them together in this section. The metal-oxide buffer for Cr-Cr_2_O_3_ can be calculated using electrochemical data from Holzheid and O’Neill (1995) and ranges from IW-6.9 at $1000~^{\circ}\text{C}$ to IW-4.4 at $1500~^{\circ}\text{C}$ (Fig. 1). A buffer for phosphorus and phosphorus oxide is not well determined, but a buffer for the reaction:

Reaction 3 $$\begin{aligned} 2\text{P} + 3\text{CaSiO}_{3} + 5/2 \text{O}_{2} = \text{Ca}_{3}(\text{PO}_{4})_{2} + 3\text{SiO}_{2} \end{aligned}$$

can be calculated from the thermodynamic data of Robie et al. (1978). This buffer ranges from IW-6.1 at $1000~^{\circ}\text{C}$ to IW-2.5 at $1500~^{\circ}\text{C}$ (Fig. 1), $\sim1\text{--}2$ log units higher than the Cr-Cr_2_O_3_ buffer. At these redox conditions, a fundamental shift in geochemical behavior occurs, with elements that normally occur with oxygen to form minerals in the crust of the Earth instead forming metal or sulfides that would potentially concentrate in the core of a planet. In the early part of the 20th century, Goldschmidt separated elements into those that occurred in the crust (lithophile), metal (siderophile) or sulfides (chalcophile) (e.g., Mason 1992). This classification is applicable to the redox state at the surface of the Earth. In contrast, the buffers for Cr and P suggest that at $\sim5$ log units more reducing than IW, elements will change their behavior. This is observed in meteorites (e.g., IAB and IIAB irons; Buchwald 1975) with the formation of the phosphide schreibersite, which can form either during crystallization of a reduced metallic magma or during later solid-state diffusive processes. In more oxidizing conditions, phosphorus typically occurs in phosphates. Similarly, chromium is typically observed under oxidizing conditions as chromite (FeCr_2_O_4_), but under reducing conditions becomes chalcophile forming daubréelite (FeCr_2_S_4_), which is a common exsolution product from troilite (FeS). These phases are observed in meteorites that formed under reducing conditions, such as enstatite meteorites, but are also found in somewhat more oxidized meteorites, like IAB irons where they coexist with silicates with modest FeO concentrations (e.g., Fa_10_, where Fa is the molar $(\text{Fe}/(\text{Fe}+\text{Mg}))*100$) in olivine and the corresponding value in pyroxene (Fs) is the molar $(\text{Fe}/(\text{Fe}+\text{Mg}+\text{Ca}))*100$. If Psyche is a core remnant rich in metal and/or sulfide, the identification of P and/or Cr as a significant component of that metal (as indicated by, e.g., a high iron concentration) would provide evidence of reducing conditions well below that of the IW buffer.

### Silicon

The Si-SiO_2_ buffer, calculated using thermodynamic data from Robie et al. (1978), marks the onset of what might be termed ultra-reducing conditions, which range from IW-14.4 at $1000~^{\circ}\text{C}$ to IW-9.7 at $1500~^{\circ}\text{C}$ (Fig. 1). Metallic silicon occurs in the Fe,Ni metal of enstatite meteorites, often comprising several weight percent. In these meteorites, silicon-bearing metal coexists with essentially FeO-free silicates. In addition to silicon becoming siderophile within enstatite meteorites, a wide range of normally lithophile elements become chalcophile, including Mg, Mn, Ti, Ca, Na, and K, forming an array of exotic sulfides including niningerite (MgS), alabandite (MnS), wassonite (TiS), oldhamite (CaS), and djerfisherite ($\text{K}_{6}\text{Na}(\text{Fe}{,}\text{Cu})_{24}\text{S}_{26}\text{Cl}$) (Keil 2010; Rubin and Ma 2017). Spectral identification of any of these phases within sulfide regions and/or identification of silicon alloyed in large metallic regions of Psyche would indicate ultra-reducing conditions.

## Key Observations

With an understanding of redox processes in general, we can address key observations to be made by the spacecraft payload to constrain the origin of Psyche. As outlined in the chapter by Elkins-Tanton et al. (this journal), revised density estimates of Psyche permit a broad range of interpretations, from a largely metallic core with significant macroporosity to a silicate-dominated assemblage similar to enstatite or CB chondrites. In this section, we first discuss the case of a largely metallic core but address how our observations are applicable to silicate-dominated cases.

### Presence of Fe,Ni Metal

The first and perhaps most robust indicator of redox state is the presence of Fe,Ni metal. Although expected, as nearly all asteroidal meteorites contain this phase, the presence of Fe,Ni metal indicates formation at a redox conditions at or below the IW buffer. The presence of Fe,Ni metal in a largely metallic asteroid could be indicated by any number of morphologic (e.g., crater rims) or spectral (e.g., reddening) features. Compositionally, iron dominates the composition of Fe,Ni metal and determination of a high percentage of iron (e.g., $>30$ wt.% Fe, based on meteorite analyses in Jarosewich 1990) on the surface of Psyche would be a strong indicator of a large metallic component. That observation might be coupled with low concentrations of elements typically bound with iron in non-metallic phases, including Mg, Si, or O. These measurements are enabled by the orbital nature of the mission, substantially improving constraints on origin compared to the pioneering flyby mission of M-class asteroid Lutetia by Rosetta (Coradini et al. 2011). Even if Fe were less than 30 wt.% and a substantial silicate component was indicated, it may still be possible to confidently determine the presence of Fe,Ni metal. McCoy et al. (2001) used elemental ratios (e.g., Fe/Si, Fe/O) constrained for individual phases and compared them to the value determined for the bulk to show that a significant fraction of Fe must be in a metallic phase on asteroid 433 Eros.

### Ni Concentration of Metal

If Fe,Ni metal comprises a significant portion of the asteroid, the Ni concentration of that metal can be a strong indicator of formation processes, including oxidation. Fractional crystallization is probably the best-known process for producing intragroup Ni variations. However, the solid metal/liquid metal partition coefficient for Ni is close to 1 ($\sim0.9\text{--}0.95$; Campbell and Humayun 2005), so the composition of the crystallizing solid only differs slightly from the composition of the liquid. This leads to a relatively restricted range of Ni concentration within any group of iron meteorites and, by extension, the common core from which they crystallized. As an example, IIIAB irons, which are the most abundant and well-studied group, range from 7.1–10.6 wt.% Ni (Wasson 1999). Condensation in the solar nebula can also produce a range of Ni concentrations in metal that is incorporated into chondritic meteorites and, later, in the cores of differentiating asteroids. In a cooling nebular gas, the first metal to condense has a Ni concentration of $19\pm3$ wt.%, which decreases with decreasing temperature (Kelly and Larimer 1977). Thus, differences in condensation temperature of the precursor chondritic meteorites that formed iron cores could, in theory, explain the range of Ni concentrations between the major groups of iron meteorites, which range from 5.3–6.5 wt.% in IIAB irons up to 16–18 wt.% in IVB irons (Goldstein et al. 2009).

While condensation could produce the range of intergroup variations observed in iron meteorites, significant evidence suggests that oxidation was the major factor in determining the Ni concentration in some groups. This would not be surprising given the relative positions of the Fe-FeO and Ni-NiO buffers discussed above. As iron is much more readily oxidized than nickel, oxidizing conditions would oxidize Fe to FeO, which would be incorporated into silicates, leaving Ni in the metallic form and enriching the metal in Ni. Consequently, an asteroid that has experienced oxidation should have a mantle and crust enriched in FeO and a core with a higher concentration of Ni. The evidence that this indeed happened comes from the study of siderophile trace elements in iron meteorites. During condensation, elements of similar volatility should be incorporated in similar concentrations, relative to the composition of the condensing gas, in metal. In contrast, during oxidation, elements that are more readily oxidized (e.g., have metal-oxide buffer curves at higher oxygen fugacity) can be fractionated relative to elements of similar volatility. This latter behavior is exactly what is observed for readily oxidized elements like W, Mo, P and Fe in some high-Ni iron meteorites (e.g., IVB irons, Campbell and Humayun 2005; Milton-South Byron trio irons, McCoy et al. 2019). Thus, a core composition enriched in Ni likely formed through oxidation. Although Fe,Ni metal indicates formation at or below the IW buffer, high Ni irons likely formed at oxidation conditions close to IW, while lower-Ni irons formed at more reducing conditions. We return later to this topic to explore in what environment and when this oxidation may have occurred.

The spacecraft’s Gamma-Ray and Neutron Spectrometer (GRNS) will provide measurements that will be used to determine the concentration and variability of elemental Ni on Psyche. The GRNS instrument design and observation plan were developed to enable detection of $\geq4$ wt% (global average) Ni content on Psyche with a one-standard-deviation uncertainty of 20% relative (Lawrence et al. this journal). This is accomplished via gamma-ray measurements of the characteristic Ni gamma rays at 1454 keV. Mapping of the Ni content on Psyche using the gamma-ray measurement is possible using summed spectra, albeit with higher uncertainty relative to the global value. Our expected statistical precision supports reporting five separate Ni concentrations, which correspond to $\sim140\text{-km-diameter}$ pixels along the equatorial ground track of the spacecraft in the lowest altitude orbit (see Chap. 1 for details). This spatial resolution is comparable to the intrinsic spatial resolution of our individual (instantaneous) gamma-ray measurements, which have a full-width at half maximum spatial resolution of 90–120 km. GRNS neutron measurements can also be used to infer the Ni content, particularly when combined with the gamma-ray measurements (Lawrence et al. 2020). These neutron data are best suited toward providing higher spatial resolution measurements of the variability of Ni across Psyche’s surface. A spatial resolution of $\sim75~\text{km}$ is possible, which will facilitate correlating Ni concentrations with large geologic units and craters. At present, at least two craters with $\sim100\text{-km}$ diameters are known to be present on Psyche (Viikinkoski et al. 2018).

### FeO Concentration in Silicates

If iron is oxidized from metal prior to or during core formation, it will be incorporated as FeO into the silicate magma that crystallizes to form the mantle and crust of the asteroid. Thus, low-Ni irons, where most of the iron remained in the core, should coexist with low FeO silicates. Conversely, high-Ni irons, where much of the iron was oxidized into the silicate shell of the asteroid, should coexist with high FeO silicates. To first order, observations of meteorites support this general relationship. As an example, the olivine-rich brachinites contain both Ni-rich metal and FeO-rich olivine (29.7 wt.% Ni, $\text{Fa}_{32.3}$ in ALH 84025; Day et al. 2012), whereas pyroxene-rich aubrites (enstatite achondrites) contain Ni-poor metal and pyroxene that is essentially FeO-free enstatite (5.8 wt.% Ni, $\text{Fs}_{0.02}$ in Aubres; Watters and Prinz 1979). In practice, many iron meteorites do not contain coexisting silicates and few, if any, groups of irons can be confidently linked to an associated group of silicate-rich stony or stony-iron meteorites. An additional complication is that while Ni fractionates little between the parent metallic magma and the crystallizing solid metal (Campbell and Humayun 2005), the same is not true for iron within the silicates. Fractionation between silicate magmas and crystallizing silicates (e.g., olivine) are well-documented, as are fractionations between different silicate minerals (e.g., olivine and pyroxene) (e.g., Righter and Drake 1997; Ruzicka et al. 1997; McCoy et al. 2006; Mandler and Elkins-Tanton 2013). Thus, silicates that do coexist with iron meteorites may not be representative of the bulk silicate magma from which they formed. As most of these silicates are likely early-crystallizing phases at the core-mantle boundary, they are likely less iron rich than the bulk of silicates crystallized from the same body. For this reason, care must be taken to constrain both the FeO concentration of the silicates and, if possible, the mineral phases present.

**Fig. 3:**
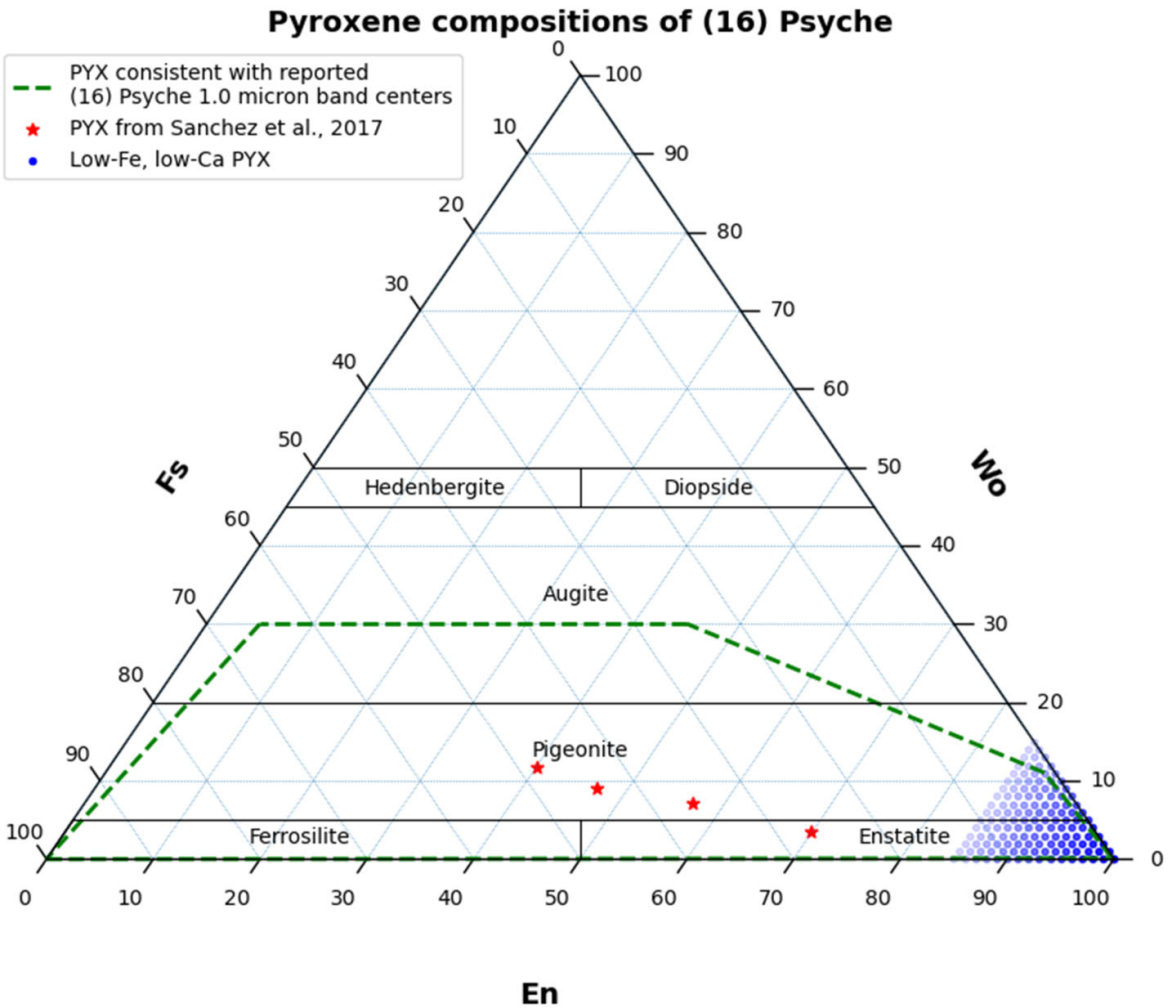
Possible pyroxene compositions on Psyche as suggested by spectroscopy of meteorites and silicates. The interpretation of Psyche as dominated by low-Fe, low-Ca orthopyroxene (blue dots) is not necessarily consistent with the range of reported band centers in a wider range of laboratory spectra of pyroxenes (red stars and green dashed field)

Mineralogical constraints on the surface compositions of asteroids can be made using remote sensing spectroscopy at UV to near-IR wavelengths ($\sim0.1\text{--}3.0~\upmu \text{m}$). These measurements reveal diagnostic spectral behavior based on comparisons to spectra of meteorites, silicates, synthetic minerals, and their mixtures (Bell et al. 2021 and references therein). For example, laboratory studies demonstrate that the band centers of $\sim1.0~\upmu \text{m}$ absorption features in reflectance spectra of Fe-bearing silicates (e.g., olivine and pyroxene) shift to longer wavelengths with increasing Fe content (e.g., Adams 1975; Cloutis and Gaffey 1991). Visible to near-IR telescopic reflectance spectra of Psyche are relatively featureless and red-sloped (reflectance increasing from $\sim10\%$ to $\sim25\%$ from 0.3 to 2.5 μm), consistent with spectra of many iron meteorites (e.g., Bus and Binzel 2002; Clark et al. 2004; DeMeo et al. 2009; Fornasier et al. 2010; Sanchez et al. 2017). Some published spectra of Psyche, though, also exhibit a broad and subtle absorption feature centered near 0.92 to 0.95 μm and varying from $\sim0\%$ to $\sim3\%$ band depth (Fornasier et al. 2010; Ockert-Bell et al. 2010; Sanchez et al. 2017). This weak feature is consistent with the presence on the asteroid of Fe-bearing pyroxenes, which are also a common constituent of metal-rich meteorites (e.g., Mittlefehldt et al. 1998). The weak band depth and relatively short-wavelength band center of this feature has been interpreted as evidence that pyroxene on Psyche occurs primarily as low-Fe, low-Ca enstatite (e.g., Fig. 3; Hardersen et al. 2011).

Laboratory spectra of both natural and synthetic pyroxenes indicate that pyroxene on Psyche could be much more Fe-rich, however. For example, Cloutis and Gaffey (1991) showed that pyroxenes with band centers between $\sim0.91$ and 0.95 μm could have between $\sim0\text{--}65$ mol.% ferrosillite ($\text{Fs} = \text{molar Fe}^{2+}/(\text{Ca}+\text{Mg}+\text{Fe}^{2+})$) and up to 30 mol.% wollastonite ($\text{Wo} = \text{molar Ca}/(\text{Ca}+\text{Mg}+\text{Fe}^{2+})$). Furthermore, Klima et al. (2007) showed that orthopyroxenes with 25 to 100 mol.% Fs also exhibited band centers between 0.913 and 0.94 μm (Fig. 3). It is thus possible that silicates on Psyche are not homogenously mixed with metal and may even be of regionally varying composition. This interpretation is consistent with whole-disk rotational spectral variations of Psyche that showed band centers ranging from 0.92 μm to 0.94 μm (Sanchez et al. 2017) and consistent with pyroxene chemistries of $\text{Fs}_{26.7}\text{Wo}_{3.5}$ to $\text{Fs}_{48.2}\text{Wo}_{11.8}$ (Fig. 3) based on analyses of HED meteorites (Burbine et al. 2007a).

High-spatial resolution mapping of Psyche at diagnostic wavelengths could therefore provide important constraints on Psyche’s silicate composition. Indeed, the narrowband near-IR filters on the spacecraft’s Multispectral Imager were chosen specifically to attempt to discriminate between low-Ca pyroxene, high-Ca pyroxene, olivine, and Fe-bearing glass by specifically characterizing the reflectance of the surface at 725, 850, 948, and 1041 nm (e.g., Dibb and Bell 2018; Bell et al. 2016, 2021). With the multispectral imaging data, these diagnostic silicates on Psyche can be globally mapped at spatial scales down to $\sim5$ meters/pixel. Although the elemental data is available on much coarser spatial scales, the optical and nuclear spectroscopy data sets can be combined to further constrain mineralogy and characterize geochemical trends (e.g., Marchi et al. 2019; Prettyman et al. 2019).

### Concentration of C, P or Si in Metal

The incorporation of carbon, phosphorus, or silicon, in that order, in iron meteorites, either as included phases or dissolved in the metallic phase, indicates increasingly reducing conditions at a temperature of $1000~^{\circ}\text{C}$ (Fig. 1).

#### Carbon

Determining the concentration of these elements within individual iron meteorites is notoriously difficult. Carbon can occur as a trace element in the metallic phase or as included graphite, which is sometimes shock-transformed to diamond or lonsdaleite, or carbides, including cohenite ($(\text{Fe},\text{Ni},\text{Co})_{3}\text{C}$), haxonite ($(\text{Fe},\text{Ni},\text{Co})_{23}\text{C}_{6}$) or the rare edscottite ($\text{Fe}_{5}\text{C}_{2}$). Many analyses of iron meteorites, particularly those done by instrumental neutron activation analysis (INAA), do not routinely include C, P or Si. Moore et al. (1969) analyzed 10–20 gram samples of iron meteorites for Ni, Co, P, C, and S. Among the 100 distinct meteorites analyzed, the Rifle mass of the Canyon Diablo meteorite – the meteorite that formed Meteor Crater in Arizona – contained the highest carbon concentration at 0.185 wt.%. However, the Rifle specimen contains a graphite nodule of 4 cm in diameter, which was not included in the analyses of Moore et al. (1969), and Buchwald (1975) and Goldstein et al. (2017) noted the presence of graphite nodules up to 5 kg within Canyon Diablo. Many low-Ni IAB irons contain abundant mixed graphite-troilite (FeS) inclusions. Thus, Canyon Diablo and some other low-Ni group IAB irons may have C concentrations that exceeded a few weight percent (Buchwald 1975; Scott and Goldstein 2012). IIAB, IIIAB and IIIE irons contain carbides and, more rarely, graphite (Scott and Goldstein 2012), consistent with lower C concentrations (Buchwald 1975).

Obtaining true bulk compositions has typically required labor- and time-intensive planimetric analyses, in which the abundance of included phases is measured on multiple large slabs, often covering areas of more than $1000~\text{cm}^{2}$. The mineralogy of each inclusion is determined optically and recombined into a bulk composition for the whole meteorite. One goal of the project’s educational student capstone projects is to automate this process using a conveyor belt photography approach followed by implementation of machine-learning algorithms. This would enable the recognition of included phases and calculation of bulk compositions (Bowman et al. this journal), substantially increasing the meteorite database for comparison to data from Psyche.

The spacecraft GRNS is based on the MESSENGER GRNS (Goldsten et al. 2007), an instrument that detected both Si and C on Mercury. Both elements are detectable via gamma-ray measurements. Detection of C via gamma-ray measurements is possible, however it is complicated by large instrument backgrounds originating from the carbon-bearing GRNS anti-coincidence shield. Peplowski et al. (2015a) reported a C concentration on Mercury of $1.4\pm0.9$ wt% C from observations of the 4438-keV gamma ray signature. There is no requirement for or formal estimate of C sensitivity for the Psyche GRNS gamma-ray measurements, however comparable performance to the MESSENGER result is likely. The most robust C measurements from MESSENGER were made with a combination of neutron, x-ray, and spectral reflectance data (Peplowski et al. 2016). That study yielded higher-precision detection of C in $\sim100\text{-km}$ deposits in three locations across Mercury’s surface. The success of a similar investigation at Psyche will depend on the chemical makeup of Psyche’s surface and the quality of elemental and mineralogical data provided by the GRNS and Imager.

A possible complication in determining the origin of carbon on Psyche’s surface is the contribution of exogenous impactors. On the howardite-eucrite-diogenite parent body, likely (4) Vesta, carbon-bearing CM2 chondrites are the most common exogenic material (Zolensky et al. 1996) and contain up to a few wt.% carbon. A useful discriminator in this respect is hydrogen, as CM2 chondrites contain abundant hydrated silicates not known from carbon-bearing iron meteorites. This approach was applied to understanding exogenic contributions to the regolith of Vesta using gamma-ray and neutron spectroscopy with the GRaND instrument on Dawn (Prettyman et al. 2012). Remote-sensing observations of Psyche have reported the presence of a spectral absorption feature associated with hydrogen, and a cross correlation of observations of Vesta and Psyche with orbital H measurements at Vesta suggests Psyche may have 200–300 ppm H (Reddy et al. 2018).

The GRNS flying on the Psyche mission is expected to have comparable performance as prior GRNS instruments. There are three means of hydrogen detection with the Psyche GRNS. For concentrations higher than $\sim0.5$ wt.%, hydrogen can be detected via the 2223-keV gamma ray (e.g., Boynton et al. 2002). For lower concentrations, like those observed at the Moon, Vesta, and expected at Psyche, epithermal neutron measurements can identify and map hydrogen concentrations as low as 50 ppm (e.g., Feldman et al. 1998; Prettyman et al. 2012; Lawrence et al. 2015). Finally, the relative fluxes of same-element gamma rays (e.g. Fe inelastic/capture ratio) and thermal neutron absoprtion can be used to measure hydrogen concentrations of $>200~\text{ppm}$ using a technique developed and applied to data from asteroid 433 Eros (Peplowski et al. 2015b).

#### Phosphorus

Phosphorus occurs in iron meteorites in solid solution within the metallic phase, as a wide variety of Ca-, Na-, Mg- and Fe-bearing phosphates (Buchwald 1984), and as the phosphides schreibersite ($(\text{Fe}{,}\text{Ni}{,}\text{Co})_{3}\text{P}$), rarer nickelphosphide ($(\text{Ni}{,}\text{Fe}{,}\text{Co})_{3}\text{P}$) and the exceptionally rare allabogdanite ($(\text{Fe}{,}\text{Ni})_{2}\text{P}$) and unnamed ($(\text{Fe}{,}\text{Ni})_{4}\text{P}$). Most iron meteorites contain less than 1 wt.% phosphorus (Esbensen and Buchwald 1979; Buchwald 1984), which is the limit for detection with the spacecraft GRNS. It is worth noting, however, that phosphorus is an incompatible element during core crystallization, and is therefore concentrated in later-forming, higher-Ni iron meteorites. Scott (1972), Buchwald (1975) and Yang and Goldstein (2005) showed strong positive correlations between P and Ni in IIAB, IIIAB, IVA and IVB irons. In the case of IIIAB irons, the later-crystallizing irons contained up to 1 wt.% P. While a general trend of faster cooling with lower Ni in IIIAB irons, indicative of early-formed, low-Ni irons occurring closer to the surface of the core, suggests inwards concentric or dendritic crystallization (Yang and Goldstein 2006), a core that experienced concentric outwards crystallization could, in theory, have a surface sufficiently enriched in P to be detectable via gamma-ray measurement, as outlined below.

An interesting phenomenon – liquid immiscibility – may occur during crystallization of a reduced, P- and S-rich magma to produce core regions with unusually high P concentrations. In this respect, IIAB and IIG irons are particularly interesting. Esbensen and Buchwald (1979) and Buchwald (1984) reported P concentrations in IIAB irons up to 0.9 wt.%. An individual analysis for the Ponca Creek mass of Ainsworth by Moore et al. (1969) contained 1.13 wt.% P, although this value is likely unrepresentative (Buchwald 1975). Chabot and Drake (2000) noted that fractional crystallization could encounter a field of liquid immiscibility in the Fe-Ni-S-P system, producing immiscible liquids with one highly enriched in S and the other moderately enriched in P. Wasson et al. (2007) argued that IIAB irons experienced liquid immiscibility with some IIAB irons containing a high fraction of trapped P-rich melt. These authors suggested that IIAB irons sample slightly less than 50% of the fractional crystallization sequence and that late-stage immiscible liquids may have separated into distinct, density separated layers, with evolution of these melts dominating the latter half of crystallization of the core. A S-rich upper layer would have crystallized greater than 90% FeS, although no meteorites apparently sampled this layer. Wasson et al. (2007) argue that these materials are more readily destroyed by erosional processes in space. Wasson and Choe (2009) argued that the six known IIG irons sample the lower P-rich immiscible liquid from crystallization of the IIAB core, an idea broadly supported by Chabot et al. (2020). A bulk P concentration of 1.7–2.1 wt.% (Buchwald 1975; Wasson and Choe 2009), if in a laterally or vertically extensive unit, might be measurable by GRNS. Further, observations of Psyche could determine the existence or fate of a hypothesized S-rich layer in such a scenario.

Phosphorous detection is possible via gamma-ray measurements. Based on a comparison of the relative gamma-ray emission probabilities and measurement efficiencies for the Si 1778-keV and P 1266-keV gamma-ray peaks, we expect that the GRNS will be sensitive to concentrations of $>1$ wt% P by the end of the primary mission (75+ days of observing time). All element detection limits, P included, can be improved through lower-altitude observations, or added observation time, which may be possible in a potential Psyche extended mission.

#### Silicon

At the most reducing conditions calculated for meteorites, silicon is incorporated into the metallic phase in enstatite chondrites. Silicon-bearing metal is inherited by related achondrites, stony-iron and iron meteorites during differentiation. Unfortunately, relatively few silicon-bearing iron meteorites exist. Wai and Wasson (1969) observed silicon concentrations below detection limits ($\sim25~\text{ppm}$) in all but two of the iron meteorites they studied sampling the nine groups known at the time, suggesting that more than 99% of iron meteorites contained less than 30 ppm Si in the metal. Silicon is found in metal particles within the highly-reduced aubrites, with average concentrations for individual aubrites ranging from 0.12 to 2.44 wt.% Si (Watters and Prinz 1979) and individual grains in aubrites exhibiting a similar range (Casanova et al. 1993). Mt. Egerton is the only well-described enstatite stony-iron meteorite and contains 4.0 wt.% Si in its metallic phase (Watters and Prinz 1979). Like a few aubrites and at least one silicon-bearing iron, Mt. Egerton also contains the nickel silicide perryite ($(\text{Ni},\text{Fe})_{5}(\text{Si},\text{P})_{2}$) as an exsolved phase from the metal (Casanova et al. 1993). Silicon-bearing irons include the impact-melted and quenched iron Nedagolla (0.14 wt.% Si) and Tucson (0.8 wt.% Si), which also contains FeO-free silicates (Buchwald 1975), as well as LEW 85369. Of special note is the Horse Creek iron meteorite, with a bulk Si concentration of 2.5 wt.%, including $\sim3$ vol.% perryite (Wai and Wasson 1969). Although few in number, collectively these samples point to the fact that the cores of ultra-reduced asteroids could contain Si concentrations measurable by remote gamma-ray spectroscopy as follows.

We expect to be able to detect $\geq2.5$ wt.% (global average) of Si concentration on Psyche with a one-standard-deviation uncertainty of 20% relative (Lawrence et al. this journal). This is accomplished via gamma-ray measurements of the characteristic Si gamma rays at 1778 keV. Mapping of the Si content will have higher uncertainty and a very coarse spatial resolution of $\sim5$ pixels over the entire asteroid. A possible complication is the occurrence of exogenous or indigenous silicates admixed with metal. The most likely source of exogenous silicates is the previously mentioned CM2 chondritic impactors (Zolensky et al. 1996). Hydrogen abundances could distinguish that contribution. Silicon dissolved in metal might be distinguished from silica in silicates by examining other elements that typically co-occur in silicates, including aluminum (feldspar), magnesium (olivine, pyroxene) and oxygen (all silicates). Al and Ca are detectable when present at concentrations greater than $\sim1$ wt.%, and the S detection limit is 3 wt.%. Mg detection is not a formal requirement of the mission, however based on comparison to unpublished MESSENGER GRS analyses, we expect to be sensitive to at least $\sim5$ wt.% Mg. Oxygen should be detectable at approximately twice the concentration of silicon, suggesting a detection limit of $\sim5$ wt.%. Fortuitously, the most common mafic silicates – olivine ($(\text{Mg}{,}\text{Fe})_{2}\text{SiO}_{4}$) and pyroxene ($(\text{Mg}{,}\text{Fe})\text{SiO}_{3}$) – have O/Si ratios of 1.7–2.3 by weight, suggesting the minimum detectable $\sim2.5$ wt.% silicon should be accompanied by $\sim5$ wt.% oxygen if bound in silicates. An O/Si ratio lower than $\sim1.7$ might indicate silicon dissolved in metal, particularly if coupled with the presence of sulfide minerals that include Mg, Mn, Ti, Ca, Na, and K. Because K is a naturally radioactive element, GRNS is sensitive to concentrations as low as 200 ppm, making this element a particularly sensitive diagnostic measurement.

### Sulfide Mineralogy

Sulfur is a ubiquitous component of iron meteorites. Highly incompatible in crystallizing metal, sulfur forms sulfides that sometimes occur as trapped or included melt within iron meteorites or, more often, is excluded during crystallization. The fate of this excluded sulfur-rich melt is highly uncertain, but sulfide-rich ($<10$ wt.% sulfide) meteorites are exceedingly rare in the world’s collection and large, sulfide meteorites are unknown. Estimates of the initial S concentration in the cores of iron meteorite groups range from 1 wt.% in IVB irons to 17 wt.% in IIAB irons (Chabot 2004) and up to 27 wt.% in low-Ni IAB irons (Goldstein et al. 2009). Within the iron meteorites themselves, Buchwald (1975) noted his planimetric analyses showed that S concentrations of 0.2–1 wt.% are quite common and above 1 wt.% not infrequent. Troilite (FeS) is the most common sulfide in iron meteorites, so much so that Buchwald (1975) specifically noted the only two sulfide-bearing irons that did not contain troilite. Daubréelite (FeCr_2_S_4_) occurs as an exsolution product from troilite, most abundantly in the low-Ni members of groups IAB, IIAB and IIIAB typically formed under reducing conditions. Interestingly, daubréelite occurs, sometimes in association with chromite (FeCr_2_O_4_), even in high-Ni IVB irons that experienced oxidation at high-temperature, leading Campbell and Humayun (2005) to argue that the redox state of the body must have changed with temperature from oxidizing at igneous temperatures to reducing at the temperature of sulfide exsolution.

As noted earlier, a wide range of normally lithophile elements become chalcophile at ultra-reducing oxygen fugacities where silicon is incorporated into metal. These include Mg, Mn, Ti, Ca, Na, and K, with an array of exotic sulfides including, most frequently, niningerite (MgS), alabandite (MnS), and oldhamite (CaS). The abundances of these phases likely to occur within a crystallizing sulfide melt are essentially unknown, as they have never been sampled. However, partial melting experiments on enstatite chondrites (McCoy et al. 1999) produced sulfide melts with 2–6 wt.% each of Mg, Ca and Cr and more than 10 wt.% Mn, suggesting these may form significant fractions during crystallization. Further, the densities of oldhamite ($2.6~\text{g/cm}^{3}$) and niningerite ($3.2~\text{g/cm}^{3}$) are significantly less than that of troilite ($4.6~\text{g/cm}^{3}$). Thus, these phases might separate by buoyancy within a crystallizing sulfide melt. Although rare, oldhamite-rich clasts, with concentrations of oldhamite up to 30 vol.%, are known from aubrites (Wheelock et al. 1994; McCoy 1998), perhaps indicative of this process.

In contrast to the metal compositions, where GRNS will likely provide the main indication of composition, sulfide mineralogy is likely best characterized by multispectral imaging. The relationship between composition and spectroscopic features exhibited by sulfides has not been characterized as well as that of the iron-bearing silicates. Most endmember diatomic sulfides (e.g., troilite – FeS; oldhamite – CaS; niningerite – MgS; and alabandite – MnS) exhibit either an absorption feature or a change in spectral slope at visible (0.3–0.7 μm) wavelengths (e.g., Britt et al. 1992; Cloutis and Gaffey 1994; Burbine et al. 2002; Helbert et al. 2013), caused by either crystal-field effects or semiconductor band-gap electronic transitions (Vaughan and Craig 1978; Cloutis and Gaffey 1994; Clark 1999).

Spectra from natural and synthetic sulfides can have markedly different absorption features, however, presumably because of differing degrees of crystallinity at microscales. For example, meteoritic troilite is characterized by a dark (less than $\sim10\%$ reflectance), red-sloped spectrum from 0.35 to 2.5 μm (Britt et al. 1992; Cloutis and Gaffey 1994), with a break to a less-red slope beyond 0.6–0.7 μm. Synthetic troilite measured by Cloutis and Gaffey (1994) has much stronger absorption features from 0.3–0.6 μm, as well as a strong $\sim1.0~\upmu \text{m}$ feature attributed to $\text{Fe}^{2+}$ crystal field effects. On the other hand, spectra of oldhamite from the Norton County aubrite exhibit strong absorption features centered near $\sim0.5~\upmu \text{m}$ and $\sim0.95~\upmu \text{m}$ (Burbine et al. 2002). Spectra of synthetic oldhamite measured by Helbert et al. (2013) only exhibit weak features near 0.55 and 0.75 μm. These distinctive absorption features of oldhamite, if present in spectra from Psyche, might provide the best evidence of formation at ultra-reducing conditions.

Several of the Multispectral Imager’s narrowband filters have been chosen to specifically attempt to discriminate between different potential sulfides on the asteroid. Specifically, measurements of the reflectance at 0.437, 0.495, and 0.55 μm could allow the detection of troilite or oldhamite on the surface, if they are present in significant quantities. A study of spectral properties of iron meteorites and their common accessory phases by Cloutis et al. (1990) indicates that the reflectance of a 90/10 wt.% mixture of iron meteorite metal and troilite powders is several percent darker than a spectrum of pure iron meteorite powder. Further work on metal-troilite mixtures by Dibb et al. (2021) shows the development of the visible wavelength absorption feature in spectra of troilite when the metal-to-troilite ratio is 60/40 wt.%. Additional evidence for oldhamite could come from detection of the narrow $\sim0.95~\upmu \text{m}$ oldhamite feature (like that in Norton County) using the Imager’s near-IR filters (e.g., Dibb and Bell 2018; Bell et al. 2016, 2021). Just like for the iron-bearing silicates, using Imager and GRNS data together could significantly help to constrain the identification of sulfides on Psyche.

## Redox Constraints on the Genesis of Psyche

The discussion above demonstrates how redox controls the mineralogy and phase compositions of an asteroidal body and, inversely, how determining the mineralogy and phase compositions can reveal the redox state of Psyche. In this section, we discuss how determining the redox state of Psyche can help us reveal the genesis of this metal-rich world.

### Meteorite Analogs as Clues to Origin

Throughout the history of asteroid spacecraft missions, links to meteorite analogs – whether argued to originate on the target asteroid or on that class of asteroids – have extended interpretations not available from spacecraft data alone. Examples include data from the Near Earth Asteroid Rendezvous (NEAR) mission suggesting links between (253) Mathilde and heated CM chondrites (Rivkin et al. 1997) and (433) Eros and ordinary chondrites (Trombka et al. 2000), data from the Dawn mission that supported Vesta as the parent body of the howardite-eucrite-diogenite meteorites (McSween et al. 2013), and orbital data from the OSIRIS-REx mission linking (101955) Bennu to hydrated CI or CM chondrites (Hamilton et al. 2019). The links strengthen meteorite-asteroid associations that can be applied to hundreds of thousands of asteroids that will never be visited by spacecraft. In addition, such links provide the distinct advantage for the mission of potentially expanding the toolset available for interpretation to mineralogical, chemical, and isotopic (including formation, impact, and exposure ages) data not attainable by spacecraft-based instruments. We will discuss several potential analogs, focusing on the how the redox state of the meteorite might further inform the redox state of Psyche.

#### Iron Meteorites

Early density estimates for Psyche (see Elkins-Tanton et al. this journal, and references therein) pointed to a metal-rich asteroid akin to the parent bodies of iron meteorites. These meteorites, composed largely of Fe,Ni metal, are commonly thought of as reduced and must have formed at or below the IW buffer. While true, iron meteorites sample a remarkable range of redox conditions. The IVB irons, enriched in Ni by oxidation and depleted in elements incorporated in metal under reducing conditions (C, P and Si), formed at an oxygen fugacity of $\sim\text{IW-1}$ (Campbell and Humayun 2005), among the most oxidized of the iron meteorites. The Ni-poor, C-rich IAB irons formed at $\sim\text{IW-2.5}$ to IW-3 (Benedix et al. 2005) likely similar to the P-rich IIAB irons (Fig. 1), intermediate among iron meteorites in redox state. This is consistent with their low Ni concentrations and incorporation of the moderately reduced elements C and P. Finally, rare Si-bearing irons, the most reduced of the iron meteorites, formed in the range of IW-5 to IW-7 if similar to Si-bearing metal in aubrites (Righter et al. 2016) to as reducing as IW-9 to IW-10 if on the Si-SiO_2_ buffer (Fig. 1), bookending a 4–9 log unit difference in redox among irons.

#### Stony-Iron Meteorites

Recent estimates of the density of Psyche between 3.4 and $4.1~\text{g/cm}^{3}$, corresponding to 30–60 vol.% metal (Elkins-Tanton et al. 2020 and references therein), expand the range of meteorite analogs. Stony-iron pallasites and mesosiderites are permissible meteorite analogs based on their density (Macke 2010).

Pallasites range from subequal mixtures of metal and silicate to metal-dominated meteorites, all with magnesian olivine of $\text{Fa}_{11\text{--}18}$ (Mittlefehldt et al. 1998). Pallasites have long been viewed as sampling the core-mantle boundary of a differentiated asteroid. Yang et al. (2010) suggested that non-uniform cooling rates require a mixed metal-silicate body formed by collision, a view which has gained increasing support.

Mesosiderites are mixtures of metal and silicates and, with only a few exceptions, silicate-dominated. There is overall agreement that impact played a role in forming mesosiderites, with their mixture of core metal and crustal silicates (Mittlefehldt et al. 1998). It remains an open question whether collisional silicate-metal mixing could produce a body of the size of Psyche with intimate mixing of metal and silicate at the cm-scale throughout the body or whether mixing would have occurred on a more localized or smaller body scale. Silicates are relatively FeO-rich (pyroxene up to $\text{Fs}_{40}$) basaltic to orthopyroxenitic clasts (Mittlefehldt et al. 1998). Viikinkoski et al. (2018) suggested mesosiderites as a meteoritic analog for Psyche, but, unlike Psyche, mesosiderite spectra exhibit deep absorption features (Burbine et al. 2007b) consistent with their FeO-rich silicates (Mittlefehldt et al. 1998).

While pallasites and mesosiderites differ in important aspects, the silicate portions of these bodies appear to have formed at similar oxygen fugacities at or near the IW buffer (Hewins and Ulmer 1984; Righter et al. 1990). The differences therefore do not result from redox state, but rather from fractionation that occurs during differentiation of the silicate mantle and crust (e.g., Righter and Drake 1997; Ruzicka et al. 1997; McCoy et al. 2006; Mandler and Elkins-Tanton 2013), a topic discussed earlier.

#### Chondritic Meteorites

Enstatite chondrites (Keil 1969) and CB, CH, and G chondrites (Weisberg et al. 1995) are permissible chondritic analogs for Psyche based on density alone (Macke 2010). From a redox perspective, these plot at a high ratio of reduced to oxidized iron on a modified Urey-Craig diagram (Fig. 2), suggesting they formed in a reducing environment.

Enstatite chondrites have essentially FeO-free silicates and up to 28 wt.% of Ni-poor (3.3–8.2 wt.%), Si-bearing metal (1.1–3.6 wt.%) (Keil 1969), indicative of formation at or below the Si-SiO_2_ buffer (Fig. 1). Enstatite chondrites would seem to be a poor match to Psyche. The FeO-free nature of their silicates is inconsistent with a weak 1 μm absorption feature and their densities marginally overlap with the low range of currently accepted densities, although we reiterate that considerable uncertainty exists in these derived values for Psyche.

CB, CH and G chondrites have slightly more iron-rich mafic silicates (olivine of Fa_3_) and up to 60 vol.% metal, with a Ni concentration of 6–12 wt.% (e.g., Weisberg et al. 1995). These meteorites illustrate the interesting point that although the compositions of their metal and silicates point to a common redox value, the metal to silicate ratios are an unreliable indicator of redox state. CB/CH/G chondrites have higher metal to silicate than enstatite chondrites but are less reduced. The high metal to silicate ratio in the former likely results from their formation as impact products, as argued by Krot et al. (2005) based on young silicate ages. It is unclear whether this high metal to silicate ratio reflects the composition of an entire asteroid or a localized region on a larger asteroid.

### The Influence of Redox-Controlled Mineralogy on Psyche

Redox is an important controlling factor in determining the mineralogy of both silicate and metal-sulfide fractions of any asteroidal body. As a result, the differentiation, solidification, and subsolidus cooling histories of these bodies can differ significantly.

#### Silicate Mineralogy

As discussed earlier, oxidation-reduction of iron between silicates and metal should result in low-Ni metal coexisting with low FeO silicates, whereas high-Ni metal should coexist with high FeO silicates. We noted that observations of meteorites support this general relationship, citing the olivine-rich brachinites (Ni-rich metal and FeO-rich olivine (29.7 wt.% Ni, $\text{Fa}_{32.3}$ in ALH 84025; Day et al. 2012)) and aubrites (Ni-poor metal and pyroxene that is essentially FeO-free enstatite (5.8 wt.% Ni, $\text{Fs}_{0.02}$ in Aubres; Watters and Prinz 1979)). A corollary to this, demonstrated by Mueller and Olsen (1967) for ordinary chondrites, is that the silicate mineralogy also changes with redox. Olivine is chemically equivalent to pyroxene with metallic iron and oxygen:

Reaction 5 $$\begin{aligned} \text{FeMgSiO}_{4} = \text{MgSiO}_{3} + \text{Fe}^{0} + 1/2\text{O}_{2} \end{aligned}$$

As a result, the chondritic precursors of asteroids will tend to be olivine-rich under oxidizing conditions and pyroxene-rich under more reducing conditions. Again, this is consistent with, brachinite formation from R chondrite-like, olivine-rich precursors (Gardner-Vandy et al. 2013), and aubrite formation from pyroxene-rich, enstatite chondrite-like precursors (Keil 2010). Of relevance to the Psyche mission is that redox controlled mineralogy should also be reflected in the ratio of, for example, silicon to oxygen, with an olivine-dominated Psyche having a lower Si/O ratio and a pyroxene-dominated Psyche a higher Si/O ratio.

#### Thermal History

Redox control of mineralogy and mineral compositions can influence thermal histories during differentiation. It is widely accepted that short-lived ^26^Al was the primary heat source for asteroid differentiation (McSween et al. 2002). The absence of reduced Al, even at the ultra-reducing conditions of enstatite meteorites, suggest that the Al-Al_2_O_3_ buffer occurs at more reducing conditions than the Si-SiO_2_ buffer and Al was not incorporated into planetary cores. Unlike aluminum, iron is redox sensitive. Reduced to its metallic form, eat-producing ^60^Fe, if incorporated into the core at the upper limit of allowable initial Solar System concentrations, could extend core solidification or slow subsolidus cooling, as suggested by Moskovitz and Walker (2011) for IVA irons. Among the possible influences of this slower cooling, formation of tetrataenite during prolonged cooling could alter the magnetic signature of Psyche, as discussed below.

#### Core and Asteroid Size

As discussed above, redox can serve as a control on the Ni concentration of the core in response to the oxidation or reduction of iron. Concomitant to this difference in Ni are differences in the size, S fraction, and crystallization temperature of the core. Bercovici (2020) calculated core radii and compositions for ordinary and carbonaceous chondrites. If we consider only those that did not experience aqueous alteration (H, L, LL, CO), core radii range from 38 to 49% of the body. Toplis et al. (2013) considered a larger range of chondrites (EH, EL, H, L, CH, CO), calculated core radii vary from 38% (L) to 55% (EH) of the body. If Psyche, with a radius of 112 km, samples a core of a differentiated body, similar core radii percentages would yield an intact parent body of 205 to 296 km in radius. These bodies would differ in volume by a factor of three and the largest of these calculated bodies would be larger than Vesta.

#### Core Sulfur Concentration

As the concentration of iron in the core changes because of oxidation, sulfur concentration should change as well. Bercovici et al. (2019) and Bercovici (2020) calculated that S varies from 9.4–21.5 wt.% of the core among H, L, LL and CO chondrites, as ƒO_2_ varies from IW-1.4 in H chondrites to IW-0.54 in CO chondrites. Thus, an oxidized Psyche might be expected to have a higher sulfur concentration in its core than a reduced Psyche with a larger core and metallic fraction. Toplis et al. (2013) calculated S concentrations of 1 wt.% (CH) to 25 wt.% (L, EL) for non-hydrated compositions. Both Bercovici (2020) and Toplis et al. (2013) emphasized that bulk composition, as much as redox, is perhaps the most significant control on S concentration in the core, as evidenced by large, S-rich cores for the CI chondrites (Bercovici et al. 2019; Bercovici 2020) and the EL chondrites (Toplis et al. 2013). Thus, while S concentration varies with oxygen fugacity, it is perhaps not the dominant control.

#### Core Crystallization

Sulfur concentration can also have a pronounced effect on the crystallization temperature of the core. In the Fe-Ni-S system (Kullerud 1963), the liquidus temperature ranges from $1539~^{\circ}\text{C}$ in the S-free case to $988~^{\circ}\text{C}$ at the Fe-FeS cotectic at 31 wt.% S. Thus, even among the non-aqueously altered chondritic compositions considered by Toplis et al. (2013) and Bercovici (2020), the liquidus temperature could vary from more than $1500~^{\circ}\text{C}$ for CH chondrites to $\sim1350~^{\circ}\text{C}$ for H chondrites to $\sim1200~^{\circ}\text{C}$ for L, LL, CO and EL chondrites. These differences might influence the onset of core formation and the duration of a molten core during asteroid cooling and crystallization. The metal-sulfide component of S-rich chondritic meteorites might be completely molten at lower degrees of silicate melting and remain molten longer during crystallization and cooling of the silicate shell. One possible outcome of this extended thermal history could be the migration of an immiscible, S-rich melt to the surface of a core stripped of its silicate shell prior to complete differentiation (Bercovici et al. 2019; Bercovici 2020), with possible detection of such S-rich materials on the surface of Psyche if it was stripped of its silicate shell while the core was still partially molten.

#### Subsolidus History

At subsolidus temperatures, redox is a major control on the Ni and P concentrations of the core and, as a result, the metallic mineralogy established during solid state exsolution. Above $912~^{\circ}\text{C}$, Fe,Ni metal, irrespective of Ni concentration, occurs as the disordered $\gamma $ phase, termed taenite in meteorites. Below this temperature, iron meteorites, with Ni concentrations of 5–60 wt.%, exsolve in the solid state to a mixture of higher-Ni, $\gamma $-Fe,Ni (taenite) and low-Ni, $\alpha$-Fe,Ni (kamacite). This transition is commonly envisioned as $\gamma \rightarrow \gamma + \alpha $ with exsolution temperatures ranging from more than $800~^{\circ}\text{C}$ at 5 wt.% Ni to $\sim600~^{\circ}\text{C}$ at 20 wt.% Ni (e.g., Romig and Goldstein 1980 and references therein). Yang and Goldstein (2005) showed that this reaction does not occur in the formation of iron meteorites. Instead, formation of kamacite is controlled by the Fe-Ni-P system with intermediate steps forming $\gamma + \text{phosphide}$, $\alpha + \gamma + \text{phosphide}$, or $\alpha _{2}$ (martensite). Irrespective of the mechanism, the relative abundance of taenite ($\gamma $-Fe,Ni) and kamacite ($\alpha $-Fe,Ni) will depend on the bulk concentration of Ni in the metallic phase, with higher-Ni irons (and the cores from which they come) having greater abundances of taenite. In addition, high-Ni irons can, if cooled slowly, form a metastable, ordered $\gamma''$ phase called tetrataenite (Yang et al. 1996). The relative abundances of these phases could potentially influence impact dynamics and, particularly, magnetics of Psyche. Microhardness measurements of taenite yield values approximately twice that of kamacite (Buchwald 1975). Thus, a greater abundance of taenite in an oxidized, high-Ni core might be more resistant to impact, although a variety of other parameters (e.g., grain boundaries per unit volume) likely also influence the response to impact (Petrovic 2001).

#### Magnetic Signature

Tetrataenite, disordered taenite and kamacite are the dominant ferromagnetic minerals in iron meteorites, which control the magnetic remanence. Tetrataenite and disordered taenite are found in μm-scale and larger rims, as nm- to μm-size grains intergrown with kamacite (plessite) and as $<200\text{-nm}$ grains embedded in a Ni-poor matrix (cloudy zone). Combined with GNRS measurements of Ni abundances, measurements of Psyche’s remanent magnetic field and/or induced magnetic field by the Magnetometry Investigation could help constrain the kamacite to taenite ratio.

The variation in grain size and abundance of taenite/tetrataenite in different iron meteorites typically leads to a $>2$ order of magnitude difference in saturation remanent magnetization (i.e., the magnetization produced after switching on and off a strong external field) between Ni-poor and Ni-rich meteorites (Dos Santos et al. 2015). Magnetic field measurements at Psyche will not provide an estimate of the saturation remanent magnetization, but will provide an estimate of the natural remanent magnetization (NRM; magnetic moment per unit mass) that these minerals may have acquired if crystallizing or cooling in a magnetic field. Both saturation and natural remanent magnetization are proportional (e.g., Wieczorek et al. 2012), which implies that taenite/tetrataenite-rich (i.e., Ni-rich) iron-meteorite-like material might be expected to carry a higher NRM than kamacite-rich (i.e., Ni-poor) material. Unfortunately, many iron meteorite samples are subject to various magnetic contaminations on Earth (hand magnets, weathering, and viscous effects from the geomagnetic field), such that this trend has not been observed in laboratory experiments (Terho et al. 1993). However, Psyche will not have been affected by such remagnetization, making magnetic measurements an additional tool to possibly constrain the Ni abundances.

The Magnetometry Investigation will also conduct electromagnetic sounding to measure the asteroid’s inductive response to the solar wind magnetic field. This method probes the internal structure of electrically conducting planetary bodies. Different induced field geometries will arise depending on the composition of the surface (and interior) of the asteroid (Elkins-Tanton et al. 2020). For example, if the oxidation state of the surface is such that iron oxides formed above the IW buffer dominate the composition, the asteroid will behave as a poor electrical conductor and have a weak induced field. This signal would likely be distinguishable from the NRM possibly carried by the iron oxides. On the other hand, a more reduced body formed at or below IW dominated by Fe-Ni will be highly conducting, such that it may be challenging to discriminate between a weak remanent field and induced field.

### Redox Constraint on Where and When Psyche Formed

#### Inner and Outer Solar System Isotopic Reservoirs

Isotopic anomalies thought to have a nucleosynthetic origin have been known from stony meteorites since the pioneering work of Clayton et al. (1973) on oxygen isotopes. Warren (2011) summarized a variety of nucleosynthetic isotopic data for chromium, titanium, nickel, and oxygen in chondrites, achondrites, stony-irons, and silicate-bearing IIIAB irons, as well as Earth, the Moon and Mars. Samples divide into two distinct populations with carbonaceous chondrites, including the metal-rich CB chondrites, forming one cluster and other meteorites (ordinary and enstatite chondrites, R chondrites, achondrites, most stony-irons, Earth, Moon and Mars) forming a second cluster. Warren (2011) argued that this bimodality may reflect episodic accretion or, alternatively, a division of material that accreted in the inner (non-carbonaceous) and outer (carbonaceous) Solar System. If the latter is true, association with a meteorite analog might provide insight on whether Psyche formed in the inner or outer Solar System.

In the case of an undifferentiated, metal-rich Psyche with low, but detectable, FeO in silicates, the inclusion of CB chondrites in the carbonaceous group might point to an outer Solar System origin. We caution, however, that the spacecraft’s instrument suite cannot measure isotopic abundances for Cr, Ti, Ni and O. Indeed, Cr and Ti concentrations will likely not be measurable remotely. Further, although the CB chondrites fall into the carbonaceous group, the petrologically-similar G chondrites are in the non-carbonaceous cluster, inferred to have an inner Solar System origin. Thus, whatever process produced these metal-rich undifferentiated meteorites operated in both isotopic reservoirs and, by extension, throughout the Solar System.

Kruijer et al. (2017) extended this dichotomy of meteorites by measuring Mo and W isotopes in iron meteorites. Like stony and stony-iron meteorites, irons also divided into two groups, termed NC and CC irons. NC irons included groups IC, IIAB, IIIAB, IIIE and IVA, whereas CC irons included IIC, IID, IIF, IIIF and IVB irons as well as the ungrouped iron Wiley. Although there were relatively few meteorite groups reported by both Warren (2011) and Kruijer et al. (2017), IIIAB irons were included in the non-carbonaceous group in both studies. Interestingly, Kruijer et al. (2017) reported that NC and CC irons differed in their two-stage Hf-W model ages, with NC iron core formation occurring $\sim0.3\text{--}1.8~\text{Myr}$ after CAI formation, whereas CC iron core formation occurred $\sim2.2\text{--}2.8~\text{Myr}$ after CAI formation. These authors argued that these two isotopic reservoirs were separated because of the formation of Jupiter $\sim1\text{--}4~\text{Ma}$ after CAI formation.

#### A Proxy for Psyche

**Fig. 4:**
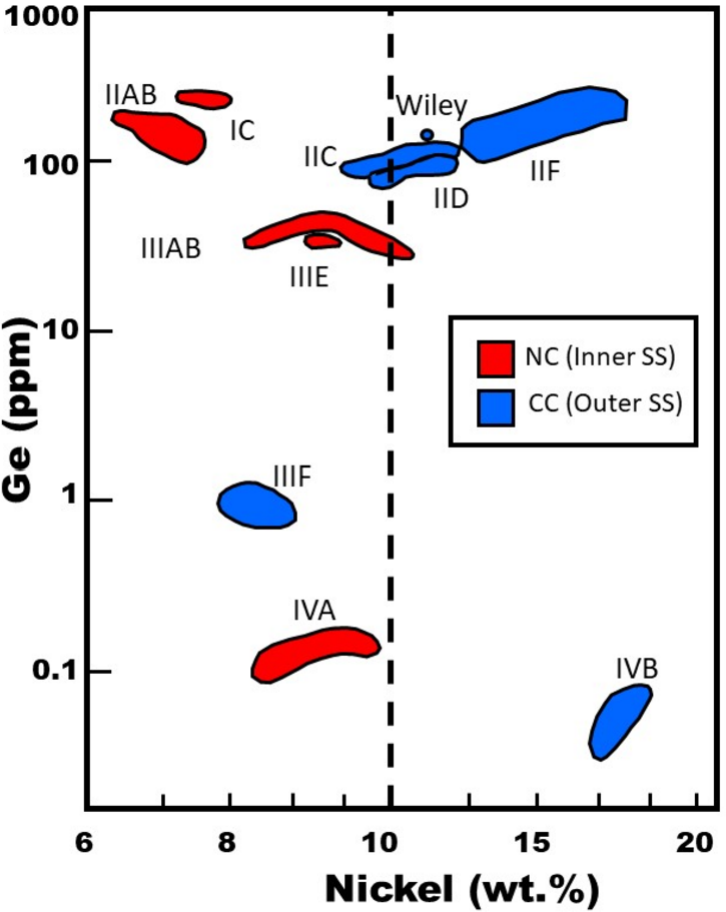
Ni (wt.%) vs Ge (ppm) for groups of iron meteorites and the ungrouped iron Wiley determined to have formed in the NC (inner Solar System) or CC (outer Solar System) isotopic reservoirs by Kruijer et al. (2017). Ni and Ge values from J.T. Wasson, as summarized in Goldstein et al. (2009) and Buchwald (1975). Excepting group IIIF irons, CC iron groups have greater than 10 wt.% Ni, while NC irons have less than 10 wt.% Ni. Thus, Ni, which is controlled in part by oxidation, may serve as a useful proxy to identify the formation location of Psyche

The instrument payload of the mission will be unable to measure Mo or W isotopes or the concentrations of these elements. Yet, the concentration of Ni may serve as a reasonable proxy for these groupings, as noted by Rubin (2018). Except for the IIIF irons, all of the groups within the CC cluster and the ungrouped Wiley have average Ni concentrations in excess of 10 wt.% (Mittlefehldt et al. 1998, and references therein). In contrast, none of the irons within the NC cluster have average Ni concentrations greater than 9 wt.% (Fig. 4). Although with somewhat greater overlap, the same holds for ungrouped irons (Spitzer et al. 2019). Thus, a Ni concentration equal to or in excess of 10 wt.% for a metal-dominated, differentiated Psyche would strongly favor an origin in the CC reservoir, presumably the outer Solar System beyond Jupiter. In contrast, a Ni concentration less than 10 wt.% would strongly, but perhaps not uniquely, favor an inner Solar System origin.

The difference in Ni concentrations between the NC and CC clusters, particularly given that these are average compositions for the various groups, almost certainly reflects oxidation of iron. The work of Kruijer et al. (2017) might provide some insights on when and where that oxidation occurred. Oxidation of Fe,Ni metal in a parent body setting would likely require interaction between metal and water, which was incorporated into the parent body as ice. The higher Ni concentrations in CC irons is, from this perspective, consistent with their origin as the cores of ice-rich asteroids accreted in the outer Solar System. However, the ages of Ni-rich CC irons present an interesting conundrum. Kruijer et al. (2017) calculated accretion times for CC iron groups, assuming chondritic Al abundances, of $\sim0.7\text{--}1.2~\text{Ma}$. These accretion times equate to 1–2 half lives of ^26^Al (720,000 years), implying a decrease in thermal energy compared to NC bodies accreted earlier. Grimm and McSween (1993) were among the earliest to suggest that accretion of ice, coupled with later accretion times in the outer Solar System, might preclude silicate melting. Castillo and Young (2016) required accretion within 1 Myr of CAIs to reach even the silicate solidus. Together, these observations might suggest that relatively late accretion of asteroids in the CC region would have prevented differentiation of ice-rich asteroids required for parent body oxidation of Fe,Ni metal to FeO. Instead, the alterative scenario is that oxidation of Fe,Ni metal occurred while still unaggregated particles in the solar nebula prior. Thus, the metal present in the CC region during late accretion of Ni-rich, CC iron meteorite parent bodies was already oxidized.

## Conclusions

The mission’s Oxidation-Reduction Working Group is focused on understanding, constraining, and applying the redox state of Psyche to decipher the origin of a metal-rich world. Specifically:

- Oxidation-reduction state of an asteroid is a key parameter in determining its history.
- The occurrence of Ni, Fe, C, Cr, P and Si, in that order, in the metal or sulfide phase of an asteroidal body indicates increasingly reduced conditions.
- Key observations made by the Imager and Gamma-ray and Neutron Spectrometer of Psyche to constrain the redox state include: the presence of Fe,Ni metal, the Ni concentration of that metal, the FeO concentration and mineralogy of silicates, the presence of C, P or Si in the metallic phase, and the mineralogy of sulfides.
- Constraining the redox state of Psyche can help constrain its origin, as well as the location and timing of its formation and possible oxidation event(s). Combined meteorite analog studies will provide a better understanding of the controls that redox has on silicate and metal mineralogy, associated differences during melting, crystallization and subsolidus cooling, and magnetic signatures.
